# Active vacuum brazing of CNT films to metal substrates for superior electron field emission performance

**DOI:** 10.1088/1468-6996/16/1/015005

**Published:** 2015-02-06

**Authors:** Rémi Longtin, Juan Ramon Sanchez-Valencia, Ivan Shorubalko, Roman Furrer, Erwin Hack, Hansrudolf Elsener, Oliver Gröning, Paul Greenwood, Nalin Rupesinghe, Kenneth Teo, Christian Leinenbach, Pierangelo Gröning

**Affiliations:** 1 Empa, Swiss Federal Laboratories for Materials Science and Technology, CH-8600 Duebendorf, Switzerland; 2 Nanotechnology on Surfaces Laboratory, Instituto de Ciencia de Materiales de Sevilla (CSIC-US), E-41092 Sevilla, Spain; 3 AIXTRON Ltd, Buckingway Business Park, Anderson Road, Swavesey, Cambridge CB24 4FQ, UK

**Keywords:** carbon nanotubes, brazing, field emission

## Abstract

The joining of macroscopic films of vertically aligned multiwalled carbon nanotubes (CNTs) to titanium substrates is demonstrated by active vacuum brazing at 820 °C with a Ag–Cu–Ti alloy and at 880 °C with a Cu–Sn–Ti–Zr alloy. The brazing methodology was elaborated in order to enable the production of highly electrically and thermally conductive CNT/metal substrate contacts. The interfacial electrical resistances of the joints were measured to be as low as 0.35 *Ω*. The improved interfacial transport properties in the brazed films lead to superior electron field-emission properties when compared to the as-grown films. An emission current of 150 *μ*A was drawn from the brazed nanotubes at an applied electric field of 0.6 V *μ*m^−1^. The improvement in electron field-emission is mainly attributed to the reduction of the contact resistance between the nanotubes and the substrate. The joints have high re-melting temperatures up to the solidus temperatures of the alloys; far greater than what is achievable with standard solders, thus expanding the application potential of CNT films to high-current and high-power applications where substantial frictional or resistive heating is expected.

## 1. Introduction

Most of the emerging and long-term potential carbon nanotube (CNT) applications [1] such as field emitters, high-current electrical interconnects, power transmission cables and thermal management in high-power applications require the availability of an appropriate joining methodology that allows the CNTs to be permanently transferred to relevant substrates leading to highly conductive, high-temperature resistant and mechanically robust contacts. Various methods of joining CNTs to each other or to other substrate materials have been attempted in the past, as outlined in the review paper of Seth Roberts and Singjai [2].

In particular macroscopic CNT films are of interest for field emitters, e.g. for application in cold x-ray cathodes [3, 4]. Brazing and soldering are the preferred joining methods for such applications.

CNT film soldering was previously demonstrated with solder alloys such as Bi–Sn–Pb [5, 6], Sn–Pb [7], Sn–Ag and Au–Sn [8]. These alloys have low melting temperatures (<280 °C) making them suitable for joining materials to electronic circuits. However from a chemical point of view, they are not appropriate for joining carbon materials. It is known since the 1960s that Cu, Ag, Au, In, Sn, Bi and Pb do not wet the surface of carbon materials like diamond and graphite [9]. Likewise, it was experimentally shown that Pb does not wet singlewalled [10] and multiwalled nanotubes [11] and that Au and Cu form discontinuous coatings on suspended CNTs [12, 13]. Alloy wetting, a necessary condition for soldering and brazing, is directly related to the strength of the interaction between the metal and carbon atoms. Reactivity to carbon is greatest for those elements having the most electron vacancies in d- and f-orbitals which rules out Au, Ag and Sn. Therefore, joining CNT films with these elements limits the joining mechanism to mechanical interlocking (nanotube entrapment) unless the CNTs are appropriately metalized with a carbide forming element. From a technical point of view, solder alloys based on Sn, Pb and In are ductile, provide limited mechanical strength and thermal stability to the joint which further discourages their use in situations where substantial heating is expected.

A well-established methodology for joining carbon based materials [14, 15] to metals is vacuum brazing with active filler alloys that contain carbide forming elements such as Ti, Zr and Cr. Diffusion of the carbide forming element towards the carbon material and the subsequent formation of an interfacial carbide, referred to as an interphase, leads to improved wetting and strong chemical bonding at the CNT/metal interface. Active brazes offer superior mechanical properties when compared to lead and lead-free solders, yet have substantially higher melting temperatures limiting the type of substrate with which they can be used. Brazing in vacuum has the advantage of preserving the excellent physical properties of the CNTs while permitting their bonding to reactive substrates such as copper and titanium by limiting both CNT and substrate oxidation. The feasibility of vacuum brazing of double-wall CNT bundles with a Ti doped Ag–Cu braze alloy was first demonstrated by Wu *et al* [16]. They confirmed the formation of strong Ti–C bonds at the CNT–braze alloy interface. However they did neither join the CNTs to metallic substrates nor test them with regard to their electrical properties.

Experimental investigations on the possibility of brazing CNT films to metals were motivated by the fact that conventional soldering cannot provide mechanically robust, conductive and high-temperature resistant contacts with substrates for applications, beyond microelectronics, aiming to exploit the excellent thermal and electrical transport properties of CNTs.

We demonstrate in this study how such films of vertically aligned multiwall CNTs can be transferred and joined to titanium substrates by active vacuum brazing. Brazing at 820 and 880 °C is demonstrated with the Ag–Cu–Ti and Cu–Sn–Ti–Zr braze alloys, respectively. The excellent wetting and spreading of the metal alloys inside the CNT is leading to strong chemical bonding and superior CNT/substrate contacts with low electrical and thermal resistances. In particular, the electron field-emission performance of the brazed CNT film is excellent and is directly related to improved interfacial electron and heat transport.

## 2. Experimental

### 2.1. CNT film synthesis

Films of vertically aligned multiwalled CNTs were synthesized from C_2_H_2_ and H_2_ by low-pressure chemical vapor deposition in a commercial reactor (Black Magic 2″, AIXTRON) at 695 °C for 20 min with a sputtered 2 nm Fe catalyst film on a 10 nm Al_2_O_3_ support layer on a high resistivity boron-doped 〈100〉 silicon substrate diced into 4 × 4 × 0.75 mm^3^ pieces.

### 2.2. Active vacuum brazing

**Figure 1.**
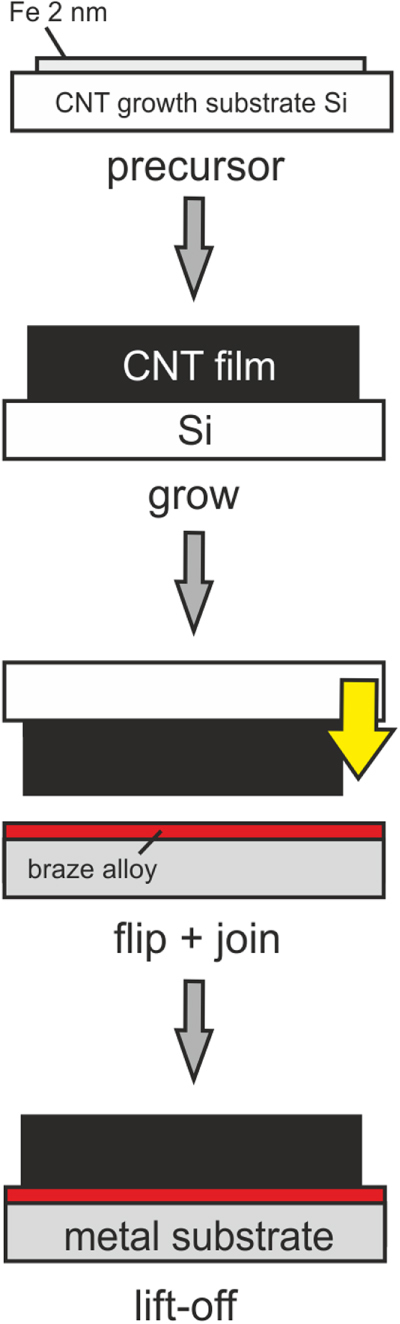
Schematic, different stages of the fabrication of active brazed CNT–metal joints.

The as-grown nanotube films were brazed facedown to 4 × 4 × 0.6 mm^3^ Ni-metalized grade 2 titanium (Ti/Ni 2 *μ*m) and to 4 × 4 × 0.95 mm^3^ grade 2 titanium substrates in a vacuum furnace (Cambridge Vacuum Engineering) at 10^−6^ mbar. The heating rate was 10 °C min^−1^, the dwell time was 5 min and the dwell temperature was 820 °C when using 100 *μ*m thick foils having a composition of Ag 63.25–Cu 35–Ti 1.75 wt% (Wesgo Metals, Hayward USA) and was 880 °C with 60 *μ*m thick foils having a composition of Cu 73.9–Sn 14.4–Ti 10.2–Zr 1.5 wt% (Sulzer Metco Germany). The solidus and liquidus temperatures for the silver alloy are 780 and 815 °C, respectively. The copper alloy has a solidus temperature of 868 °C and a liquidus temperature of 925 °C [17]. The brazing foils were made by mixing a metal alloy powder (325 mesh: particle size <44 *μ*m) with an organic binder. The resulting paste was manually printed on a flat surface, dried in air and compressed into a foil to the desired thickness. The braze foil, substrate and inverted CNT film are assembled in a jig and held in place with an adjustable screw during brazing. Once the brazing step was completed, the Si substrate was removed with tweezers. For inspection, the joints were manually cleaved transversely and longitudinally with a steel blade. The different stages of the process are sketched in figure 1.

### 2.3. Characterization methods

A FEI ESEM-FEG XL-30 scanning electron microscope operated at 20 kV was used to examine the CNT joints. A Carl Zeiss Orion Plus helium ion microscope (HeIM) was used for high resolution imaging. HeIM allows imaging of samples with a surface resolution of 0.3 nm and has a different contrast mechanism than electron microscopes [18]. This improved resolution is necessary to reveal structural details at the nanometer scale over larger, more representative areas in a non-destructive way as opposed to transmission electron microscopy. The typical parameters for image acquisition were: 30 kV of acceleration voltage, beam currents of 0.5–1 pA, a dwell time of 2 *μ*s with a line averaging of 16–32.

A CRM200 WiTec confocal Raman microscope equipped with a 10× (0.25 NA) objective with a 532.3 nm laser set at 5 mW in combination with a 600 grooves/mm grating was used to track changes in nanotube graphitization after brazing.

Contact pads (2 nm Cr/200 nm Au) were deposited on one side of the joints, via shadow masking in a Plassys II electron beam evaporator system, with the following geometry: 100 *μ*m in width, 1 mm in length, spacing within the same material was 100 *μ*m and spacing across the joint was 500 *μ*m. A Keithley 2001 electrical characterization equipment in combination with a closed four-probe station were used to obtain current–voltage curves across the joints in the dark and at room temperature.

The field-emission properties over a 4 × 4 mm^2^ area of the brazed films were measured at base pressures of 10^−7^ mbar with a scanning anode field-emission microscope (SAFEM) [19]. The joints were mounted on a horizontal *x*–*y* translation stage. The anode consisted of a spherical tip 1 mm in radius mounted on a cantilever that was moved in the *z*-direction in 100 nm steps. The emission current was measured with a Keithley 237 source-measure unit at a fixed anode-to-sample distance of 500 *μ*m.

## 3. Results and discussion

### 3.1. Structure and morphology of brazed CNT–substrate joints

**Figure 2.**
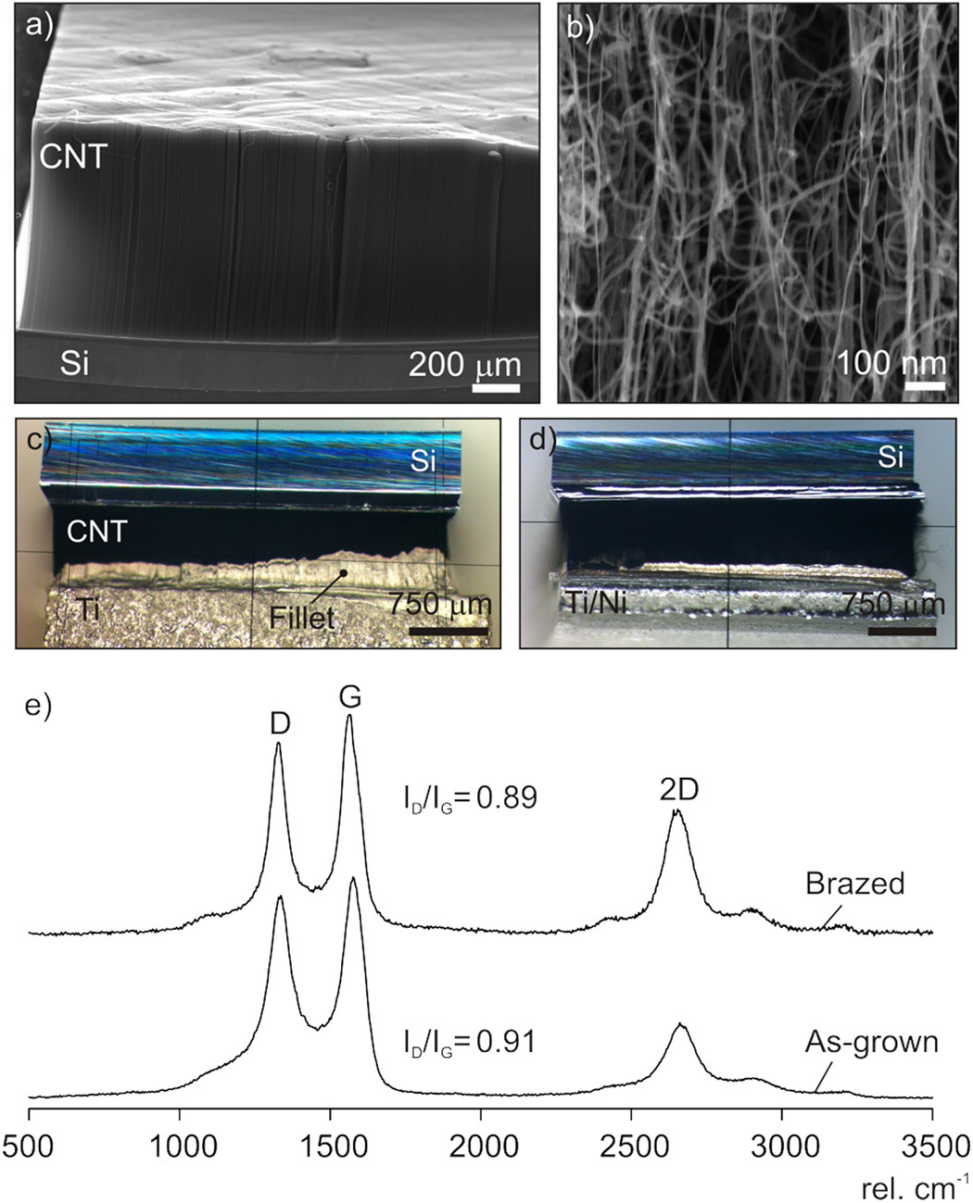
(a) SEM image of a multiwalled carbon nanotube film on silicon prior to brazing. (b) High magnification HeIM image of the CNTs. Optical microscope image of CNT films brazed to (c) Ti and (d) to Ni-metalized Ti (Ti/Ni) at 880 °C with the Cu–Sn–Ti–Zr filler alloy. (e) Raman spectra of the surface of the as-grown and brazed CNT films indicating a slight increase in graphitization after brazing.

A typical CNT film with a density of 10^10^–10^11^ CNTs cm^−2^ grown on silicon is shown in figure 2(a). The vermicular nanotube diameters range from 2 to 20 nm as seen by HeIM in figure 2(b). Two representative CNT films brazed to Ti and Ti/Ni substrates with the Cu–Sn–Ti–Zr alloy at 880 °C are shown in figures 2(c) and 1(d) respectively. In both cases, the braze alloy has formed a fillet along the film’s edge which is indicative of a chemical reaction leading to wetting.

Raman spectra of the top surface of the as-grown and brazed film after silicon lift-off are shown in figure 2(e). The Raman spectra indicate a slight increase in graphitization [20] after brazing. The G peak width decreased from 77 to 58 cm^−1^ and the 2D peak width decreased from 123 to 114 cm^−1^. The intensity ratio of the D to G peaks (*I*_D_/*I*_G_) also decreased from 0.91 to 0.89. A similar decrease in this ratio was reported when annealing multiwalled CNTs in vacuum above 800 °C [21].

**Figure 3.**
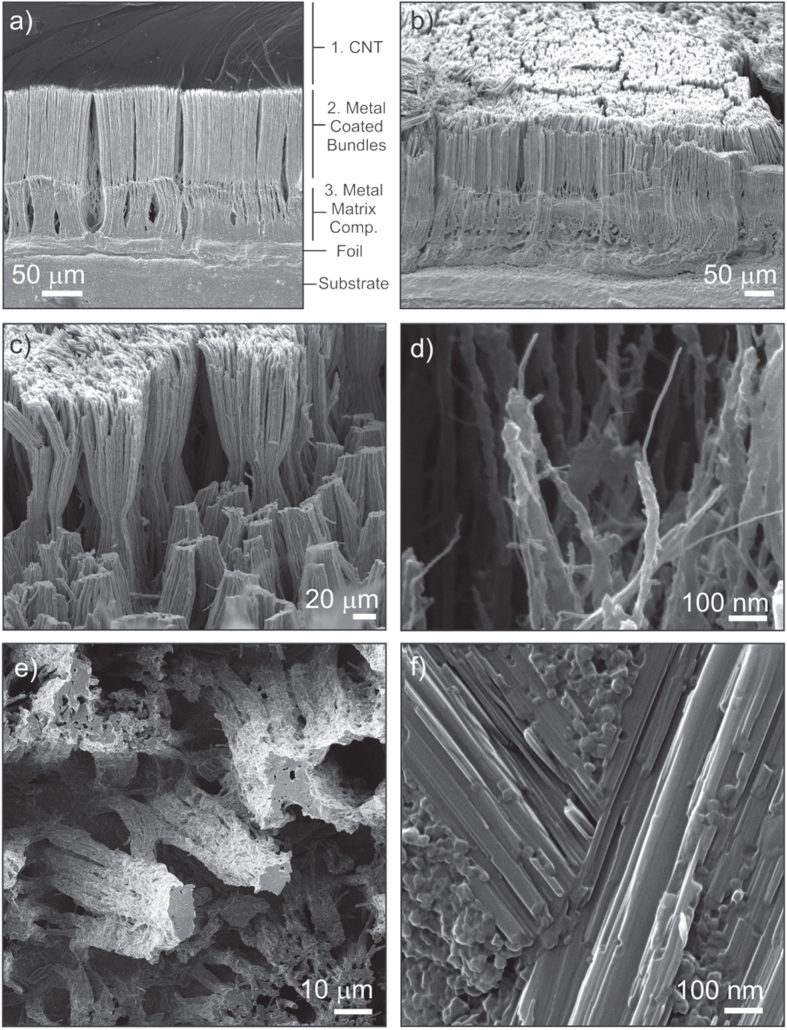
(a) Side view SEM image of the Cu–Sn–Ti–Zr fillet with labeled regions. (b) SEM image (55° tilt) of the top of region 2 after the removal of the CNT layer in region 1. (c) SEM image (55° tilt) of the bundles in region 2. (d) Side view HeIM image of the top of region 2 showing individual CNTs coated with metal. (e) Top view HeIM image of the fractured metal matrix composite bundles. (f) High magnification HeIM image of a composite bundle’s fracture surface.

The side view SEM image of the Cu–Sn–Ti–Zr fillet, after Si lift-off, reveals three distinct regions as shown in figure 3(a). Region 1 at the top of the film consists of CNTs having retained more or less their vertical alignment after brazing. Region 2 contains metal-coated CNT bundles while the region closest to the brazing foil is characterized by larger bundles completely encased in metal; hereafter referred to as the metal matrix CNT composite region. The partially melted brazed foil is seen below this region and above the substrate.

Brazing is usually carried out above the liquidus temperature of the filler alloy at 925 °C, however preliminary experiments have shown that this alloy, when it is fully liquid, excessively penetrates the CNT film and reacts with the Si substrate preventing lift-off. At 880 °C, 90% of the alloy is liquid which is sufficient for joining while limiting the infiltration to the first ∼100 *μ*m. The top CNT layer (region 1) was mechanically removed with a blade as shown in figure 3(b). This image reveals how the molten alloy infiltrated the lower portion of the CNT film by capillarity. The bundling pattern observed in region 2 and shown in figure 3(c) is consistent with the so-called nanocarpet effect which is caused by lateral capillary forces during the invasive spreading of a liquid inside an ordered array of high aspect ratio structures [22, 23].

The combination of shear and bending forces during the removal of the CNTs in region 1 lead to two fracture planes: at the bundle waist and between regions 1 and 2 (figure 3(c)). A high magnification HeIM image of the protruding CNTs in region 2 is shown in figure 3(d). Individual metal-coated CNTs can be resolved here. The fractured metal matrix composite bundles are shown in figure 3(e) and a HeIM image of the fracture surface is shown in figure 3(f). Individual CNTs can no longer be resolved here even at high magnification. Rather, flat crystals embedded in a matrix of irregular particles are seen in figure 3(f).

**Figure 4.**
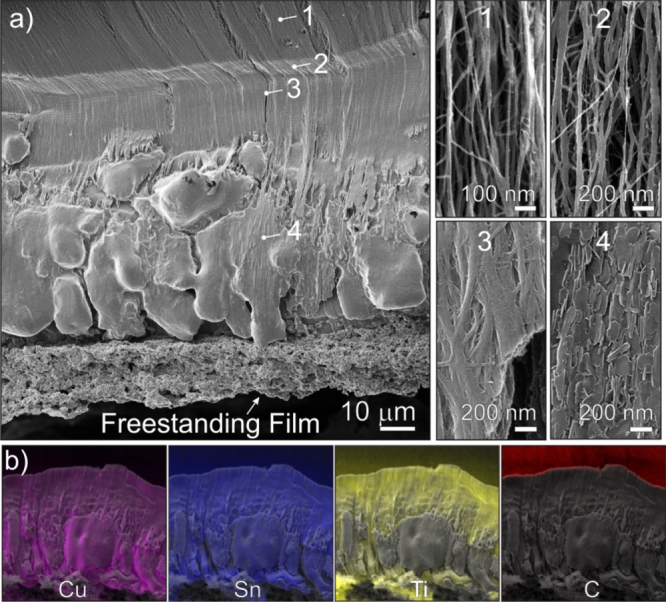
(a) HeIM images of different regions along the joint’s transverse cross-section: (1) nanoparticles on CNTs (region 1), (2) metal-coated CNT (top of region 2), (3) partially encased bundles (bottom of region 2), (4) metal matrix composite (region 3) (b).

High magnification HeIM images of the different regions along the joint’s transverse cross-section, obtained by mechanical cleaving, are also shown in figure 4(a). These images confirm that the different regions observed along the fillet are also distinguishable in the interior of the film. Nanoparticles are seen on the aligned CNTs in region 1 far from the joint line. Individual CNTs and small bundles thereof are coated with metal at the top of region 2. Partially encased bundles are identified in the lower part of region 2. The fracture here is due to shear forces during cleaving. The metal matrix composite containing flat hexagonal crystals is seen in region 3. The qualitative results of an EDX elemental mapping of a selected area between regions 1, 2 and 3 are shown in figure 4(b). While Cu and Ti are clearly enriched in the lower part (i.e. in the composite region), a slight Ti enrichment can be also seen in the CNT region in the upper part. This indicates the strong tendency of Ti to interact with the CNTs.

Figures 2 and 3 reveal that the joint microstructure is anisotropic with a complex metallurgy. It arises from the interaction of a quaternary alloy with a porous carbon material at high temperatures. It is difficult to characterize in detail and at the nanoscale the metallurgy of the joint since differences in solid state atomic surface diffusion, on the CNTs’ outer graphene walls, and liquid state diffusion lead to elemental segregation. Chemical reactions away from equilibrium condition will occur locally and over short time scale leading to the formation of various compounds such as stoichiometric and sub-stoichiometric carbides as well as intermetallic phases, based on the Cu–Sn, Cu–Ti and Sn–Ti binary systems, as were experimentally identified [24] and predicted by thermodynamic assessments of the Cu–Sn–Ti system [25].

A detailed characterization of the microstructure is beyond the scope of this work, yet it is evident that the improved wetting of the CNTs in region 2 is due to the formation of a carbide interphase between the alloy and the outer CNT walls. Indeed, a thin reaction layer of TiC was experimentally observed at the CNT/Ag–Cu–Ti interface after brazing at 1000 °C [16]. Likewise, Chen *et al* observed the formation of a Ti_*x*_C layer on single wall CNTs ultrasonically bonded to Ti electrodes [26], Similarly, the presence of a 5 nm SiC interphase on CNTs was confirmed experimentally and was credited with the improved wetting of an Al alloy containing 23 wt% Si [27]. Concerning region 3, the solubility of C in Cu is extremely low, in the parts per million range [28], making it unlikely that the CNTs were completely dissolved as atomic carbon in the melt. It is possible that the CNTs were fully converted to carbide particles since the thickness of the TiC layer that is formed when brazing diamond under similar conditions is larger than the diameter of the CNTs.

**Figure 5.**
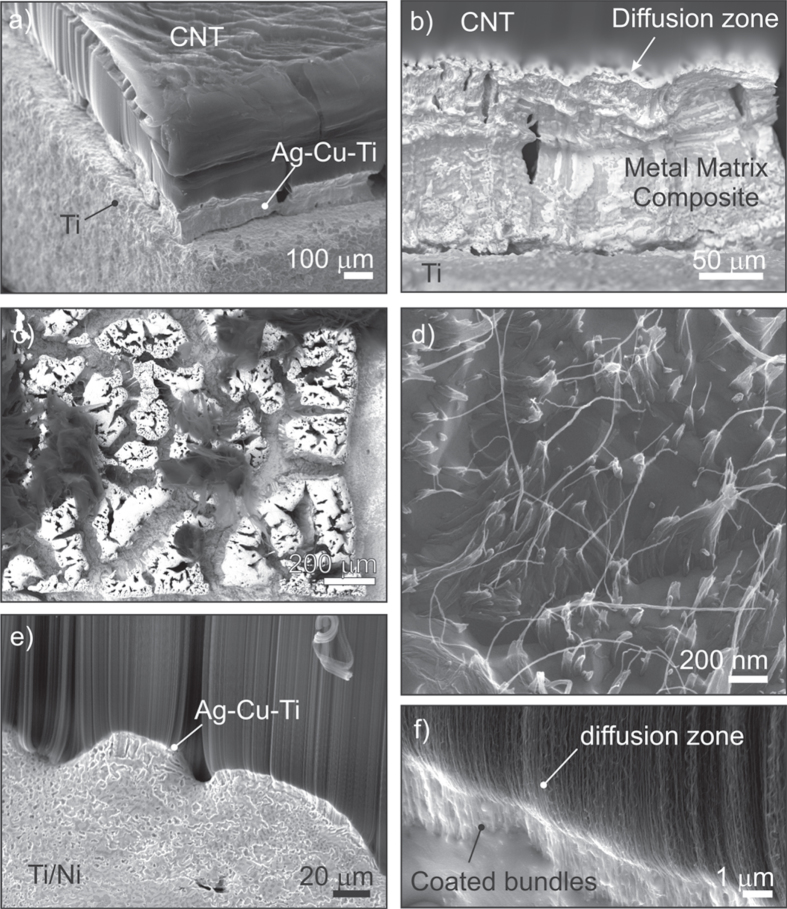
(a) SEM image of a CNT film brazed to Ti with the Ag–Cu–Ti alloy. (b) SEM image of the fillet showing the metal matrix composite region, the diffusion zone and the aligned CNTs. (c) SEM top view image after removal of the top CNT layer. (d) High magnification HeIM image of the top of a metal matrix composite bundle showing metal-sheathed nanotubes protruding from the matrix. (e) SEM image of the fillet when brazing CNTs on Ti/Ni with the Ag–Cu–Ti alloy. (f) SEM image of the diffusion zone and coated bundles.

A second alloy, Ag–Cu–Ti, containing only 1.75 wt% of Ti was used to join CNT films to Ti and Ti/Ni substrates at 820 °C, that is, above the liquidus temperature of this alloy. A typical CNT film brazed to Ti after silicon lift-off is shown in figure 5(a). A fillet is seen on the edge of the CNT film similarly to what was observed for the Cu–Sn–Ti–Zr braze, however the metal matrix composite region is now separated from the top CNT region by a thin diffusion zone as shown in figure 5(b). Cu and Ag especially are known to be highly mobile on graphene. Again, the bare CNTs in region 1 were removed mechanically and revealed extensive bundling leading to a porosity of ∼48% as shown in figure 5(c). A high magnification HeIM image of the top of one of the metal matrix bundles reveals individual metal-sheathed CNTs protruding from the matrix (figure 5(d)). Evidently, the CNTs were not fully converted to TiC here. This is due to the reduced Ti content and lower brazing temperature. Slight microstructural differences are observed when brazing CNTs on Ti/Ni. The fillet height is reduced and bundling is less pronounced with the metalized substrate (figure 5(e)). Furthermore, a region a few micrometers in length with metal-coated bundles is now seen below the diffusion zone (figure 5(f)). Additional EDX elemental mappings led to very similar results as in the case of brazing with the Cu–Sn–Ti–Zr alloy.

Overall, the CNT brazing process with the Cu–Sn–Ti–Zr and Ag–Cu–Ti alloy, respectively, can be described as follows: as the temperature is progressively raised above the solidus temperature, the brazing alloys will begin to melt and the Ti will start reacting with the CNTs to form a TiC interlayer. The resulting liquid will spread along the CNTs on this interlayer as well as laterally into the film leading to bundling. Solidification close to the substrate will lead to the formation of a metal matrix composite. The metal atoms that have diffused on the surface of the CNT walls from the braze foil into region 1 will eventually coalesce into nanoparticles. No significant difference, apart from fillet height, was remarked when brazing CNTs to the bare and metalized substrates with this alloy.

### 3.2. Electrical and field emission properties

**Figure 6.**
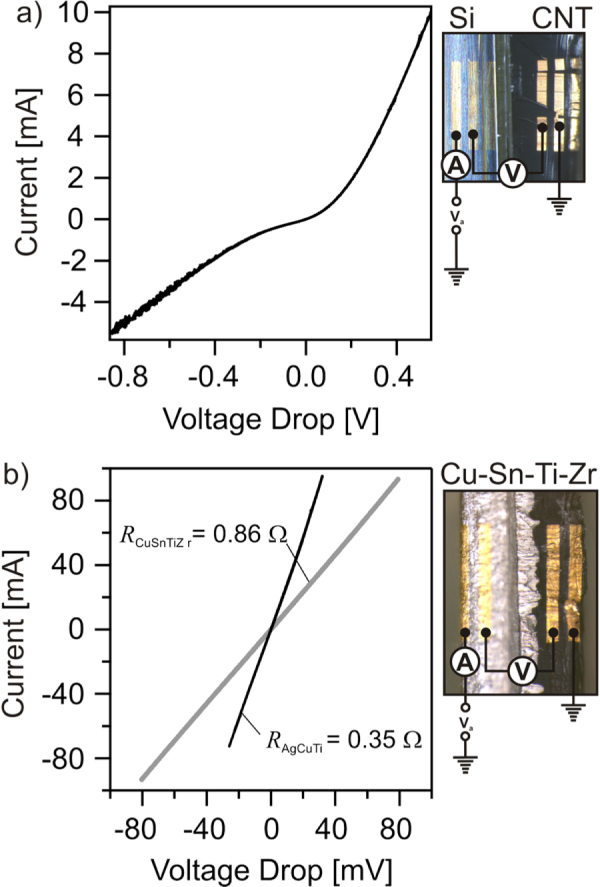
(a) Four-probe current versus voltage curves of the (a) Si/CNT interface and (b) across the Cu–Sn–Ti–Zr and Ag–Cu–Ti joints.

It was demonstrated that both alloys can be used to join CNT films to titanium substrates. The joint properties were measured to confirm the applicability of such assemblies. The electrical resistances across the joints were determined by four-probe electrical measurements. Two gold contact pads were produced on the side of the CNT film (region 1) while the other two were on the substrate. Two probes were used to supply current while the other two measured the voltage drop across the joint. The results are shown in figure 6 with schematic representations of each measurement. The current versus voltage (*I*–*V*) curve across the Si/CNT interface for the as-grown film is provided in figure 6(a). The nonlinearity of the *I*–*V* curve in combination with the polarity of the applied bias is consistent with a Schottky diode-like junction consisting of a *p*-doped Si substrate and metallic CNTs. Fitting the linear portion of the curve yield a resistance of 40 *Ω* with a positive voltage and 125 *Ω* with a negative voltage. The *I*–*V* curves for the brazed films are shown in figure 6(b). The linearity indicates an ohmic contact with the substrate across both joints. The Ag–Cu–Ti joint shows slightly lower resistance of 0.35 *Ω* than the Cu–Sn–Ti–Zr joint with 0.86 *Ω*. The electrical conductivity for the Ag–Cu–Ti alloy is 23×10^6^ *Ω*^−1^ m^−1^ according to the supplier while conductivity values of ~7×10^6^ *Ω*^−1^ m^−1^ are typical for bronzes with 11 wt% Sn [29]. It is clear that the presence of the braze alloy significantly reduces the contact resistance between the nanotubes and the substrate when compared to when they are grown on Si.

Again, the presence of the braze layers improves the interfacial transport properties by reducing the thermal contact resistance when compared to CNTs grown on Si. The nonlinear temperature profile in the CNT film is indicative of an anisotropic solid with varying physical properties. This is consistent with the anisotropic microstructure observed.

So far, the joints were shown to possess superior interfacial transport properties when compared to the as-grown CNT films on Si. One application that would clearly benefit from low electrical and low thermal resistance contacts is CNT cold electron sources. It was recently demonstrated how thermionic electron sources in commercial x-ray tubes can be replaced by CNT-based cathodes to produce x-rays without requiring any further modification to the device design [3]. In spite of this demonstration, several challenges remain and limit the widespread use of CNTs as cold electron sources. The maximum current that can be drawn per emitter and the contact resistance between the CNTs and the substrate were identified as the most crucial parameters affecting macroscopic emission behavior [30]. It is possible to reduce the contact resistance by employing metallization layers between the nanotube growth catalyst and the Si substrate and by carrying out post-treatments on the emitters [30]. Brazing is another approach to reduce emitter contact resistance, as demonstrated in this work.

**Figure 7.**
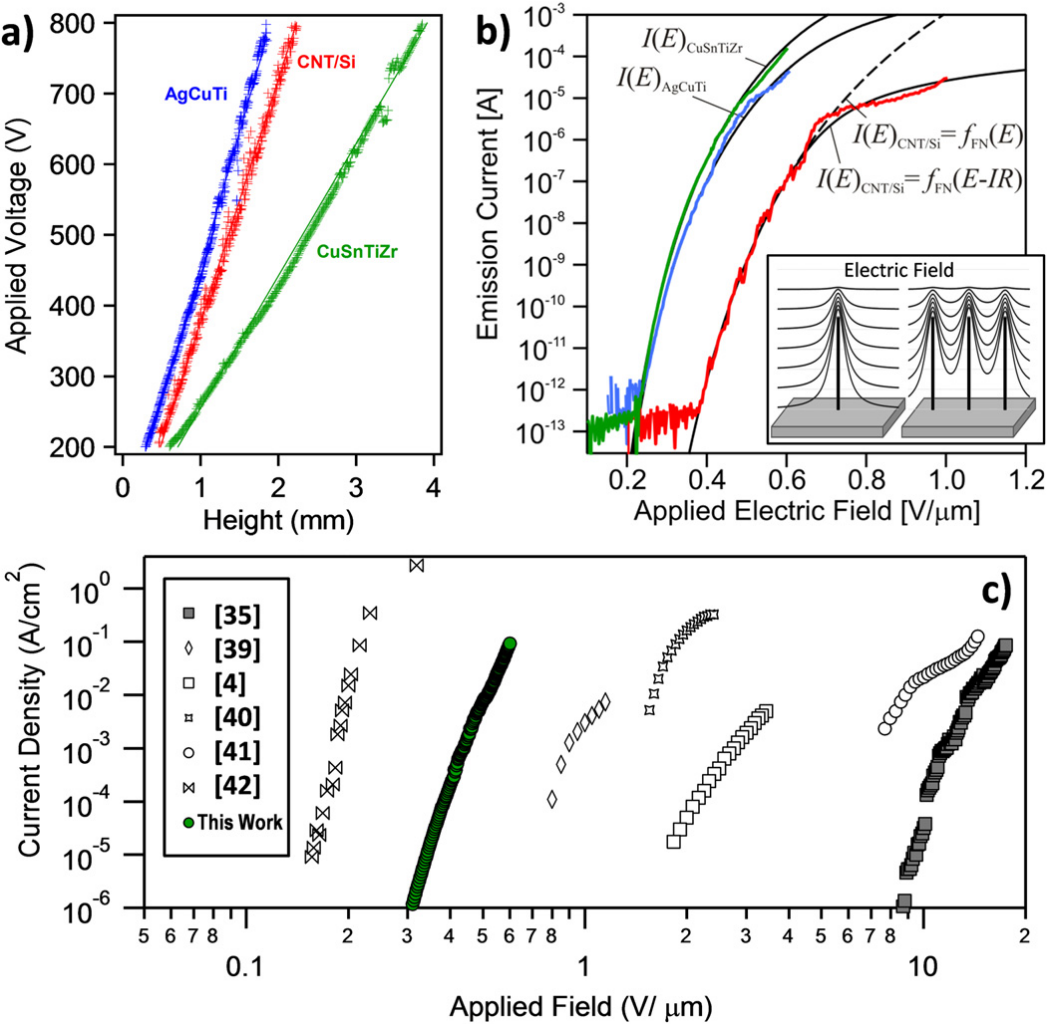
(a) Applied voltage versus anode-CNT distance and (b) field-emission current versus applied electric field for the brazed CNT film on Ti/Ni and for the CNT film grown on Si. (b) The ideal emitter behavior is described by the FN model according to: *I*(*E*) = *f*_FN_(*E*) (dashed line). The contact resistances can be obtained from the resistor-limited FN fits according to: *I*(*E*) = *f*_FN_(*E* − *IR*) (solid lines). (c) Literature comparison of emission current density versus applied electric field with the results obtained in this work.

The field-emission behavior of the brazed CNT films on Ti/Ni was measured with a SAFEM and compared to the emission of a CNT film grown on Si. The instrument allows an accurate determination of the CNT apex height by means of the voltage versus anode-CNT distance plots which are shown in figure 7(a). From the resulting linear plot, the location of the emitter apex can be extrapolated as the height for *V* = 0. This is a very important aspect, since the real anode-CNT apex distance can be accurately determined for every measurement, obtaining a direct measurement of the applied electric field. In addition to the CNT height determination, the slope of the curve gives information related with the so called field enhancement factor (*β*) caused by the accumulation of the electric field lines at the CNT apex due to their high aspect ratio (see inset in figure 7(b)). The *β* value for an individual CNT is uniquely related with the geometry of the emitter and can be calculated in first approximation (i.e. floating sphere model) from the equation *β* = *h*/*r*, with *h* and *r* the height and radius of the CNT, respectively. However, dense CNT forest samples present drastically reduced *β* values due to the screening from neighbor tubes (inset in figure 7(b)) which emission is usually limited by randomly distributed ones that stick out from the sample. The determination of *β* can be calculated from the slope of the voltage versus anode-CNT distance curves assuming that the electric field needed at the CNT apex to achieve an emission current of 50 nA is around 4000 V *μ*m^−1^ [31]. It is remarkable that the slopes obtained from the *V* versus anode-CNT distance are very low (between 0.38 and 0.2 V *μ*m^−1^) giving rise to extremely high *β* values ranging from around 10 000 to 20 000. Such high values are obtained for both brazed and as-grown CNT with a radius of around 10 nm as determined by SEM images in figure 2. The calculated *β* indicates that the height of tubes which stick out from the forest surface is around 100–200 *μ*m, which is in good agreement with the SEM images.

The emission current (*I*) versus applied electric field (*E*) plots are presented in figure 7(b)) and were recorded after several cycles applying a maximum field of 0.6 V *μ*m^−1^. After several measurements, stable and reproducible curves were obtained. For the case of the as-grown CNT, a significant and continuous degradation was observed after every measurement. Due to that, the applied field was increased to a maximum of 1 V *μ*m^−1^ to reach significant emission currents. The emission behavior of the CNTs on Si is consistent with the well-known Fowler–Nordheim (FN) model (*I*(*E*) *= f*_FN_(*E*)) that describes the ideal emitter behavior up to currents of around 1 *μ*A. The deviation from the FN model can be explained by considering the presence of a voltage drop along the nanotube representing a resistance, at the nanotube/substrate interface, which is in series with the emitter. The data can be fitted by solving numerically: *I*(*E*) = *f*_FN_(*E* − *IR*) where *R* is the contact resistance parameter [32]. This is referred to as the resistor-limited FN fit. An equivalent resistance of 4 M*Ω* is obtained from the curve in figure 7(b)) for the CNTs grown on Si which is consistent with the values of 5 M*Ω* previously reported [32]. A much lower contact resistance of 10 k*Ω* is obtained for the CNTs brazed with the Cu–Sn–Ti–Zr alloy and 100 k*Ω* is obtained for the Ag–Cu–Ti joint. It should be noted that the resistance values extracted from the correction to the FN characteristic cannot be directly compared to the measured electrical resistances since the modified FN relation expresses the link between a voltage drop and a change in field-enhancement [21].

The turn-on field for a detectable emission of 0.5 pA is reduced from 0.4 V *μ*m^−1^ for the CNTs on Si to 0.2 V *μ*m^−1^ for the brazed films. The field-enhancement factors, that can be estimated from the emitters’ height-to-radius aspect ratio, can also be extracted from the FN fits [21] and are around 5800 for the as-grown film and 11 000 for the brazed CNT films. The initial beta values extrapolated from the voltage versus anode-CNT distance are higher than the ones calculated from the FN fit. This is likely caused by a partial degradation of the tubes due to the high current achieved during the measurements. A maximum current of 150 *μ*A at 0.6 V *μ*m^−1^ was drawn from the Cu–Sn–Ti–Zr brazed nanotubes and 42 *μ*A for the Ag–Cu–Ti braze. Only 0.1 *μ*A was drawn from the as-grown sample at this field while 30 *μ*A was obtained at 1 V *μ*m^−1^. Individual CNT emitters typically provide maximum 10–100 *μ*A [30] and can be pushed to yield up to 120 *μ*A when annealed in vacuum [21]. We thus conclude on the basis of the measured current that only a limited number of high field-enhancement emitters, randomly distributed over the cathode area contribute to the measured currents in figure 7(b)). An accurate determination of the current density would require knowledge of the exact location of the dominant field-emitters. Although the current density provided by the CNTs cannot be calculated, the area measured with a 1 mm diameter spherical tip is around 0.0016 cm^2^ [3]. This indicates that the minimum current density provided is around 93 and 26 mA cm^−2^ for the Cu–Sn–Ti–Zr and the Ag–Cu–Ti brazed samples, respectively. Figure 7(c) shows some representative current density versus applied electric field curves obtained from the literature and the Cu–Sn–Ti–Zr brazed sample [3, 6, 33–36]. From the comparison with the literature it can be concluded that the brazed samples studied here present outstanding field emission properties among which the following can be highlighted.

(i) Extremely high field enhancement noticeable by the low turn-on field (ca. 0.2 V *μ*m^−1^). To our knowledge, the lowest turn-on field has been reported by Hazra *et al* [36], with similar values to the brazed samples reported here.
(ii) Very low contact resistance between CNT and the supporting metal that allows emission currents higher than 0.093 A cm^−2^. Such high currents are usually obtained by structuring the samples (like the work reported by Chiu *et al* [34]). In this work, similar current densities are obtained for the brazed CNT due to the improved metal–CNT contact.

The improvement in emitted current results mainly from the improved contact with the substrate which reduces the electrical resistance and promotes heat dissipation away from the CNT/substrate interface. This allows the nanotube emitters to be operated at higher currents before the onset of degradation. The power dissipated at the base of the nanotube at a mere 1 *μ*A can reach several hundreds of W cm^−2^. Resistive heating can lead to substrate melting and explosive damage of CNT bundles as was experimentally observed [30]. The slight increase in nanotube graphitization during brazing may also have contributed to improve the emission current by reducing the intrinsic resistance of the nanotubes. Although out of the scope of this work, we are convinced that the combination of the brazing technique developed here with catalyst structuring will be ideal candidates for field emission applications.

As a final remark, the fact that the nanotubes are brazed rather than soldered leads to joints with high re-melting temperatures. The joints will retain their integrity at temperatures at least up to the solidus temperatures of the braze alloys used. This directly translates into the possibility of using the brazed films as components in devices that require harsh downstream processing steps such as vacuum sealing for commercial x-ray source manufacturing carried out at 780 °C [3]. More importantly, braze alloy contacts allows for operating CNT devices at performance levels previously unachievable due to the inability of low melting point solder contacts, especially indium alloy contacts, to cope with the heat generated during device operation.

## 4. Conclusions

The joining of macroscopic films of vertically aligned multi wall CNTs to bare Ti and Ni-metalized Ti substrates was demonstrated by active vacuum brazing at 820 °C with the Ag–Cu–Ti braze and at 880 °C with the Cu–Sn–Ti–Zr braze. The formation of a TiC interphase on the nanotubes is credited for the wetting and spreading of the filler alloy inside the porous nanotube film, leading to a mechanically strong bond. The resulting joint microstructures are anisotropic with complex metallurgies involving the formation of carbides, intermetallic phases and solid solutions. Brazing leads to a slight increase in nanotube graphitization and to low electrical and thermal resistance contacts with the substrate which greatly improve the electron field-emission properties.

The described brazing methodology is applicable for joining macroscopic CNT films to several other substrate material such as steel, copper and nickel. Moreover, it prevails the vertically aligned CNT structure which is important for the field emission properties. This greatly expands the application potential of CNT beyond vias and electrical interconnects. The brazed CNT films could make excellent cold electron cathodes for x-ray sources or could be alternative materials to graphitic foams and carbon–carbon composites for thermal management applications in various land, space and aerospace applications. The joints have high re-melting temperatures; at least up to the solidus temperatures of the respective filler alloys, which means that they can survive most processing steps required for e.g. encapsulation or vacuum-tight sealing of x-ray sources.
